# Lactate dehydrogenase predicting mortality in patients with aneurysmal subarachnoid hemorrhage

**DOI:** 10.1002/acn3.51650

**Published:** 2022-08-19

**Authors:** Xin Zan, Haidong Deng, Yu Zhang, Peng Wang, Weelic Chong, Yang Hai, Chao You, Fang Fang

**Affiliations:** ^1^ Department of Neurosurgery West China Hospital, Sichuan University Chengdu Sichuan China; ^2^ Center for Evidence Based Medical Affiliated Hospital of Chengdu University Chengdu Sichuan China; ^3^ Department of Medical Oncology Thomas Jefferson University Philadelphia Pennsylvania USA; ^4^ Sidney Kimmel Medical College, Thomas Jefferson University Philadelphia Pennsylvania USA

## Abstract

**Objective:**

Lactate dehydrogenase (LDH) has been reported to be associated with outcomes after surgery in patients with aneurysmal subarachnoid hemorrhage (aSAH), but it is unclear if this is independent from other biomarkers and across all aSAH treatments. This study aims to assess whether LDH is an independent predictor of mortality in patients with aSAH and test whether the inclusion of LDH in a well‐established prediction model can improve discrimination and reclassification.

**Methods:**

This was a retrospective observational study at a tertiary academic medical center. This study measured baseline LDH levels taken at admission and longitudinal LDH levels (up to a month postadmission) to assess median, max, and trajectory LDH levels. The primary outcome was mortality at 90 days. Multivariable regression analyses were used to evaluate associations between LDH and outcomes. The full original Subarachnoid Hemorrhage International Trialists' (SAHIT) model was used as the reference model.

**Results:**

In total, 3524 patients with aSAH were included. LDH at admission was independently associated with mortality at 90 days (quartile 4 vs. 1: odds ratio 1.60; 95% CI 1.08–2.37) and mortality at the longest follow‐up (quartile 4 vs. 1: hazard ratio1.72; 95% CI 1.34–2.20). Compared with the SAHIT model, the addition of three LDH (admission, max, and median) levels to the SAHIT model significantly improved the area under the curve and categorical net reclassification improvement for prediction mortality.

**Interpretation:**

In patients with aSAH, LDH level is an independent predictor of all‐cause mortality. The incorporation of LDH into a well‐established prediction model improved the ability to predict the risk of death in patients with aSAH.

## Introduction

Aneurysmal subarachnoid hemorrhage (aSAH) is a devastating form of stroke, with significant morbidity and mortality.^1^, ^2^ Identifying high‐risk patients based on clinical characteristics is essential to perform interventions to improve survival.^3^, ^4^, ^5^, ^6^ A major drawback is that there are few available models calibrated for clinical practice.^7^ One widely accepted and validated tool is the Subarachnoid Hemorrhage International Trialists (SAHIT) model.^8^ It was developed from 10,936 patients and validated in external validation with 3355 patients. The full model of SAHIT includes age, neurological grade on admission, premorbid hypertension, clot volume, aneurysm size, aneurysm location, and treatment. Only 22%–31% of the variation in outcome is explained by these variables in the SAHIT model, indicating that other factors have substantial effects on the outcome or that outcome scales are imprecise. To improve on this, one solution is to recalibrate the original SAHIT model with additional predictors.

Serum lactate dehydrogenase (LDH) is a low‐cost and routine laboratory test conducted as part of hospital admission. Thus, it could potentially be an ideal predictor for clinicians to determine the prognosis for patients with aSAH without additional cost. LDH is a cytoplasmic enzyme expressed in all major organs, including the liver, heart, lung, and brain.^9^ Serum LDH levels are considered a marker of inflammation and reflect the degree of brain tissue injury.^10^, ^11^, ^12^, ^13^, ^14^, ^15^ Recent studies indicate that LDH is associated with postoperative pneumonia^16^ and functional outcome^17^ in patients with aSAH after surgery. However, it is unclear if LDH level is an important predictor for mortality, in addition to other factors (C‐reactive protein and glycemia) and across all aSAH treatments, and if adding LDH to the SAHIT model can improve outcome prediction.

In the present study, we aim to investigate the association between serum LDH and mortality in patients with aSAH and to evaluate the value of LDH in risk stratification of mortality beyond that of the original SAHIT model.

## Methods

### Study design and data source

We conducted a retrospective, observational study focusing on the clinical characteristics of confirmed cases of aSAH in West China Hospital Sichuan University, a tertiary academic medical center, between January 2009 and June 2019. All patients with aSAH were identified via a search of the electronic medical records. West China Hospital Institutional Review Board approved the study, with a waiver of informed consent. Patient care was standardized according to the prevailing guidelines on the care of aSAH patients at the time.^1^, ^2^

### Patient selection

Patients diagnosed with aSAH through the following were included: subarachnoid hemorrhage diagnosed via head computed tomography, magnetic resonance imaging, or angiography, and an aneurysm confirmed with cerebral angiography, magnetic resonance angiography, or computed tomography angiography.

Participants were excluded if they met any of the key exclusion criteria: (1) aneurysms caused by trauma or arteriovenous malformations, (2) fusiform aneurysms, and nondefinitive aneurysms; aneurysms were treated before ictus or (3) lack of admission LDH. We also excluded patients whose identification numbers in the electronic medical record system were wrong or nonexistent or their household registration was not in Sichuan province.

### Variables and outcome

The predictor assessed is a serum LDH level, with blood samples collected from patients within 24 h after admission and before treatment of aneurysms. In this hospital, the LDH test was usually obtained at admission and was repeated every 1–4 days by the clinicians. We obtained serial LDH measurements for a month after admission, and thus, this study further assessed the impact of trajectory, median, and max LDH levels. All patients were divided into four groups according to LDH levels as quartile. In sensitivity analysis, patients were divided into a high‐level group and a low‐level group according to the clinical threshold of LDH level (333 U/L).^18^

The primary outcome was mortality at 90 days. Secondary outcomes were mortality at 180 days, mortality at 1 year and at the longest long‐term follow‐up, and in‐hospital complications (including pneumonia, intracranial infection, urinary tract infection, bloodstream infection, hydrocephalus, rebleeding, delayed cerebral ischemia, and seizures).

### Follow‐up

All death records were extracted through the Household Registration Administration System. This system is based on the Seventh National Census which was completed in 2020. In China, the law mandates that, if a citizen dies, the head of household, relatives, dependents, or neighbors shall report the death registration to the household registration authority and cancel the household registration within 1 month. Thus, this system has accurate death records.^19^ Therefore, the rate of loss to follow up of this study was negligible.^20^ For all participants, the median follow‐up was 6.6 years, the longest follow‐up period was 12.5 years, and the censoring date was August 15, 2021.

### Statistical analysis

Descriptive statistics were conducted to summarize baseline characteristics, with numbers and percentages for categorical variables and means (SD) for continuous variables. ANOVA and chi‐square test were computed as appropriate for between‐group comparisons. Missing values were replaced with the mean for continuous values and others for categorical variables.

Logistic regression models were performed to analyze unadjusted and adjusted associations between outcomes and LDH levels in each group. Covariates in the logistic regression models were derived from covariates examined in prior studies and clinical expertise,^1^, ^7^ including age, sex, smoking, alcohol abuse, hypertension, diabetes mellitus, coronary heart disease, chronic obstructive pulmonary disease, chronic renal failure, aneurysm location (anterior circulation, posterior circulation), the size of aneurysm, time from onset to admission, systolic blood pressure, blood glucose, troponin T, neutrophil count, procalcitonin, C‐reactive protein, Hunt & Hess grade, Fisher grade, external ventricular drain, and operation of aneurysm (no treatment, clip, and coil). These factors were calculated by univariable binary logistic regression. Factors associated with the outcome (*p* < 0.10) were included in a multivariable binary logistic regression model: variables were considered independent when they remained statistically significant. Kaplan–Meier curves and log‐rank tests of the time to event data were used to mortality at the longest follow‐up.

We additionally performed propensity score matching to control confounding variables as a secondary analysis. Logistic regression as the method was chosen to calculate propensity score matching, and 1:1 nearest neighbor matching without replacement with a caliper width of 0.25 SD was performed. Standardized mean difference biases were computed to ensure balance after propensity score matching between groups, and a difference of <0.1 was considered to be well balanced. We also performed multivariable logistic regression with the LASSO method, which simultaneously selects the variables.

In addition, we performed subgroup comparisons between patients with low LDH levels at admission (<189 U/L) and high LDH levels at admission (≥189 U/L) to evaluate for differences in mortality at 90 days in patients with aSAH.

We assessed improvement in model performance for the addition of LDH levels to the SAHIT model by calculating the change in the area under the curve (AUC), net reclassification improvement (NRI), relative integrated discrimination improvement (IDI), as recommended by the TRIPOD statement.^21^, ^22^ A comparison of AUC was performed using the DeLong test. Calibration was evaluated graphically with calibration plots and statistically by computing a goodness‐of‐fit test of the model using the Hosmer‐Lemeshow test.

No exploratory statistical analyses were performed. Significance was set at a *p* value of <0.05, and all *p* values were two‐sided. All statistical analyses were performed with R software (version 4.0.5; Foundation for Statistical Computing).

## Results

A total of 3524 patients with aSAH were included in this study (Fig. S1 in the Appendix). Baseline characteristics of patients are presented in Table 1. Patients with higher LDH levels were significantly more often women, having higher odds of hypertension, external ventricular drain and were more likely to present with higher Hunt & Hess grades and Fisher grades. They have also presented a higher biomarker concentrations trend. There were no statistically significant differences in alcohol abuse, diabetes, coronary heart disease, chronic obstructive pulmonary disease, chronic renal failure, aneurysm location, and size of aneurysm of patients with aSAH between groups (*p* < 0.05).

**Table 1:** Admission characteristics stratified by baseline lactate dehydrogenase levels.

Characteristics	Lactate dehydrogenase quartile (U/L)				
	Q1 < 161, *N* = 898	Q2 161–189, *N* = 867	Q3 190–228, *N* = 881	Q4 > 228, *N* = 878	*p*
Demographics					
Age, year, mean (SD)	51.77 (11.78)	54.98 (11.67)	57.03 (11.71)	56.76 (11.99)	<0.001
Female, *n* (%)	356 (39.6)	285 (32.9)	296 (33.6)	293 (33.4)	0.007
Smoking					
Current	41 (4.6)	42 (4.8)	43 (4.9)	27 (3.1)	0.002
Ever	216 (24.1)	155 (17.9)	182 (20.7)	154 (17.5)	
Never	641 (71.4)	670 (77.3)	656 (74.5)	697 (79.4)	
Alcohol abuse, *n* (%)	201 (22.4)	155 (17.9)	177 (20.1)	162 (18.5)	0.077
Medical history, *n* (%)					
Hypertension	191 (21.3)	195 (22.5)	225 (25.5)	272 (31.0)	<0.001
Diabetes	55 (6.1)	41 (4.7)	52 (5.9)	58 (6.6)	0.389
Coronary heart disease	22 (2.4)	16 (1.8)	26 (3.0)	23 (2.6)	0.506
Chronic obstructive pulmonary disease	54 (6.0)	55 (6.3)	65 (7.4)	75 (8.5)	0.155
Chronic renal failure	1 (0.1)	4 (0.5)	9 (1.0)	7 (0.8)	0.068
Systolic blood pressure, mean (SD)	138.58 (22.36)	142.76 (22.81)	148.71 (24.34)	149.27 (28.54)	<0.001
Aneurysm characteristics					
Anterior location, *n* (%)	142 (15.8)	158 (18.2)	176 (20.0)	176 (20.0)	0.072
Size of aneurysm, cm, mean (SD)	0.77 (0.74)	0.72 (0.61)	0.74 (0.68)	0.78 (0.73)	0.323
Fisher grade, *n* (%)					
I	55 (6.1)	47 (5.4)	24 (2.7)	26 (3.0)	<0.001
II	147 (16.4)	142 (16.4)	129 (14.6)	124 (14.1)	
III	117 (13.0)	113 (13.0)	116 (13.2)	69 (7.9)	
IV	289 (32.2)	325 (37.5)	402 (45.6)	492 (56.0)	
Hunt & Hess grade, *n* (%)					
I	108 (12.0)	110 (12.7)	74 (8.4)	55 (6.3)	<0.001
II	566 (63.0)	504 (58.1)	436 (49.5)	327 (37.2)	
III	180 (20.0)	194 (22.4)	259 (29.4)	269 (30.6)	
IV	41 (4.6)	54 (6.2)	103 (11.7)	196 (22.3)	
V	3 (0.3)	5 (0.6)	9 (1.0)	31 (3.5)	
External ventricular drain	14 (1.4)	11 (1.2)	26 (2.6)	32 (3.3)	0.002
Operation of aneurysm					
No treatment	98 (10.9)	122 (14.1)	140 (15.9)	207 (23.6)	<0.001
Clip	696 (77.5)	641 (73.9)	627 (71.2)	536 (61.0)	
Coil	104 (11.6)	104 (12.0)	114 (12.9)	135 (15.4)	
Baseline biomarker, mean (SD)					
Neutrophil count, ×10^9^/L	6.98 (3.37)	7.91 (3.71)	9.38 (4.23)	10.97 (5.09)	<0.001
Procalcitonin	0.18 (0.32)	0.59 (2.31)	0.27 (0.46)	2.22 (9.56)	0.003
C‐reactive protein	29.98 (48.93)	46.45 (71.23)	39.24 (51.61)	76.43 (89.89)	<0.001
Blood glucose, mmol/L	6.36 (1.97)	6.72 (2.15)	7.19 (2.42)	7.67 (2.86)	<0.001
Troponin T, ng/L	14.40 (36.00)	48.44 (161.82)	43.30 (91.62)	131.26 (246.51)	<0.001

Table 2 exhibits unadjusted and adjusted associations between quartile of LDH levels and mortality at 90 days. In univariate regression, the 90 days mortality rate was significantly higher in patients in the Q4 of LDH at admission than in patients in the Q1 (OR, 4.40, 95% CI 3.19–6.08). In multivariate regression, the association remained significant for patients in the Q4 compared with patients in the Q1 (OR, 1.60, 95% CI 1.08–2.37), after adjusting for age, COPD, chronic renal failure, systolic blood pressure, aneurysm location, size of aneurysm, troponin T, blood glucose, neutrophil count, C‐reactive protein, Hunt & Hess grade, Fisher grade, external ventricular drain, and operation of aneurysm (Table S1 in the appendix). In logistic regression with LASSO penalty (Table S2 in the appendix), compared with Q1 mortality at 90 days, the Q4 OR was 1.48 (95% CI 1.02–2.14). There was a strong and continuous relationship between LDH at admission and mortality at 90 days and the proportion of death was increased according to LDH at admission (Fig. S2 in the appendix). When LDH was analyzed as continuous, the adjusted OR of 90 days of mortality for each 1‐point increase in LDH after logarithmic transformations was 1.98 (95% CI 1.30–3.02, Table S6). When LDH was divided by the clinical threshold of LDH level (333 U/L),^18^ the adjusted OR was 1.63 (95% CI 1.05–2.53) clinical threshold of LDH levels, and mortality at 90 days. The propensity‐matched analysis for the association between LDH at admission and 90 days mortality remained significant.

**Table 2:** Unadjusted and adjusted associations between quartile of LDH levels and mortality at 90 days.

LDH measured time	LDH levels (U/L)	Events, *n* (%)	Unadjusted OR	Multivariable regression adjusted OR^1^	Propensity score matching adjusted OR^2^	LASSO adjusted OR^3^
Admission	<162	52/898 (5.8%)	1 [Reference]	1 [Reference]	1 [Reference]	1 [Reference]
	162–189	64/867 (7.4%)	1.30 (0.89–1.89)	0.91 (0.60–1.40)	0.86 (0.55–1.34)	1.01 (0.67–1.51)
	190–228	105/881 (11.9%)	2.20 (1.56–3.11)	1.06 (0.70–1.61)	1.16 (0.75–1.78)	1.18 (0.81–1.73)
	>228	187/878 (21.3%)	4.40 (3.19–6.08)	1.60 (1.08–2.37)	1.78 (1.16–2.73)	1.48 (1.02–2.14)
Median	<169	38/886 (4.3%)	1 [Reference]	1 [Reference]	1 [Reference]	1 [Reference]
	169–202	61/894 (6.8%)	1.63 (1.08–2.48)	1.04 (0.64–1.70)	1.12 (0.70–1.81)	1.22 (0.79–1.90)
	202–247	107/863 (12.4%)	3.16 (2.15–4.63)	1.42 (0.90–2.24)	1.40 (0.88–2.23)	1.69 (1.11–2.56)
	>247	202/881 (22.9%)	6.64 (4.63–9.52)	2.54 (1.65–3.92)	2.08 (1.28–3.35)	2.11 (1.40–3.18)
Max	<186	43/897 (4.8%)	1 [Reference]	1 [Reference]	1 [Reference]	1 [Reference]
	186–232	72/878 (8.2%)	1.77 (1.20–2.62)	0.87 (0.53–1.44)	1.14 (0.73–1.78)	1.18 (0.78–1.81)
	233–305	111/870 (12.8%)	2.90 (2.02–4.19)	1.59 (1.02–2.46)	1.46 (0.94–2.27)	1.63 (1.08–2.44)
	>305	182/879 (20.7%)	5.19 (3.67–7.34)	2.33 (1.51–3.59)	2.25 (1.43–3.54)	2.05 (1.37–3.07)

LASSO, least absolute shrinkage and selection operator; LDH, lactate dehydrogenase; OR, odds ratio.

^1^ Variables associated with the outcome (*p* < 0.10) in univariable binary logistic regression were included in a multivariable binary logistic regression model. This model adjusted for age, chronic obstructive pulmonary disease, chronic renal failure, systolic blood pressure, aneurysm location, size of aneurysm, troponin T, blood glucose, neutrophil count, C‐reactive protein, Hunt & Hess grade, Fisher grade, external ventricular drain, and operation of aneurysm.

^2^ This model adjusted for age, sex, smoking, alcohol abuse, hypertension, diabetes mellitus, coronary heart disease, chronic obstructive pulmonary disease, chronic renal failure, aneurysm location (anterior circulation, posterior circulation), the size of aneurysm, time from onset to admission, systolic blood pressure, blood glucose, troponin T, neutrophil count, procalcitonin, C‐reactive protein, Hunt & Hess grade, Fisher grade, external ventricular drain, and operation of aneurysm.

^3^ Multivariable logistic regression with LASSO strategy, which simultaneously selects the variables. This model adjusted for chronic renal failure, size of aneurysm, Fisher grade, Hunt & Hess grade, external ventricular drain, neutrophil count, and glucose.

Median/max LDH levels during hospital were also associated with 90 days of mortality. Similar results of mortality at 180 days, mortality at 1 year, mortality at 2 years, and the longest follow‐up are shown in Table S3 in the appendix.

**Figure 1:**
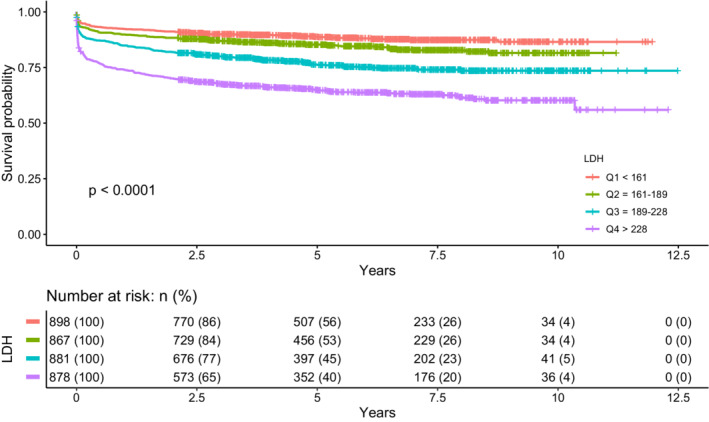
Kaplan–Meier survival curves indicating the relationship between lactate dehydrogenase at admission and all‐cause mortality after aneurysmal subarachnoid hemorrhage during a median follow‐up of 6.6 years.

Kaplan–Meier survival analysis illustrated higher LDH level was associated with worse long‐term survival over the follow‐up period (*p* < 0.001; Fig. 1). During the 12.5 years, after adjustment variables, higher LDH level was associated with a significantly increased all‐cause mortality (quartile 4 vs. 1: hazard ratio 1.72; 95% CI 1.34–2.20).

**Figure 2:**
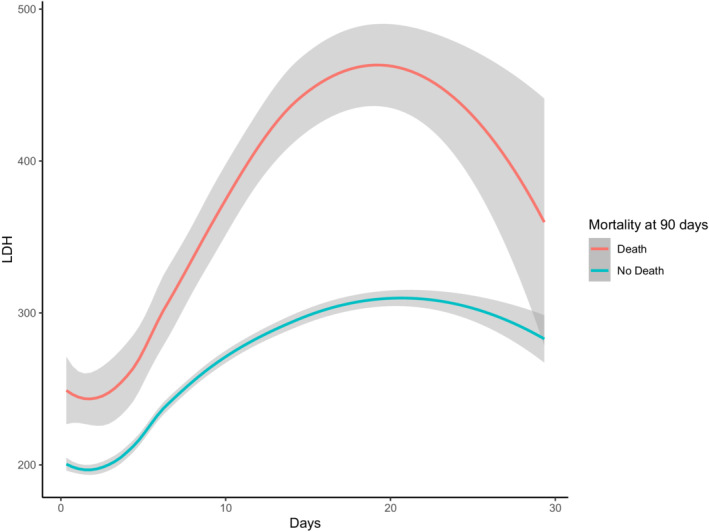
Trajectory of lactate dehydrogenase levels over time among patients with aneurysmal subarachnoid hemorrhage.

During hospital admission within 30 days, a total of 2794 had serial LDH measurements, and 1848 patients have measured LDH more than four times. The peak median LDH levels was 294 U/L, measured on hospital day 17. Higher peak LDH levels were reported among patients who died within 90 days than in survival patients (*p* < 0.001, Fig. 2).

The associations with in‐hospital complications are summarized in Table S4. Pneumonia developed, urinary tract infection, bloodstream infection, delayed cerebral ischemia, and seizures were significantly higher in patients in the Q4 than in patients in the Q1.

We further assessed interactions by variables on different LDH levels (Fig. S3 in the appendix). In multivariable analysis, the interaction term for age and Hunt & Hess grade was statistically significant. (*p* for interaction <0.05).

Compared with the original SAHIT model, all three adding LDH (admission, median, and max) showed an improved AUC (Table 3 and Fig. S4 in the appendix), with ∆AUC of 0.01 (*p* = 0.01), 0.02 (*p* < 0.001), and 0.02 (*p* < 0.001). We further investigated reclassification, and the results were along the same line. Categorical NRI was significant in all three adding LDH models. Continuous NRI was also significant in adding the admission LDH model, median LDH model, and max LDH model. The Hosmer‐Lemeshow test confirmed the goodness of fit for all models (Fig. S5 in the appendix).

**Table 3:** Discrimination, reclassification, and Calibration measures of the models.

Models	Discrimination			Reclassification		Calibration
	AUC (95% CI)	∆AUC (*p*)	IDI, % (*p*)	Categorical NRI, % (*p*)	Continuous NRI, % (*p*)	Hosmer‐Lemeshow *p*
Original SAHIT model	0.80 (0.78–0.83)	Reference	Reference	Reference	Reference	0.34
+ Admission LDH	0.81 (0.79–0.83)	0.01 (0.01)	0.8 (0.005)	3.9 (0.017)	14.8 (0.004)	0.89
+ Median LDH	0.82 (0.80–0.84)	0.02 (<0.001)	1.1 (0.005)	5.5 (0.004)	25.8 (<0.001)	0.42
+ Max LDH	0.82 (0.79–0.84)	0.02 (<0.001)	0.8 (0.03)	4.1 (0.006)	39.2 (<0.001)	0.36

AUC, area under the curve; IDI, integrated discrimination improvement; LDH, Lactate dehydrogenase; NRI, net reclassification improvement; SAHIT, Subarachnoid Hemorrhage International Trialists.

## Discussion

In this large cohort study, we demonstrated that LDH was associated with mortality after aSAH, independent of conventional predictors, and novel inflammatory biomarkers. Moreover, the addition of LDH significantly improved the discrimination and reclassification capability of the SAHIT model.

There are available data on serum LDH levels in relation to poor outcomes after aSAH. Ding et al.^16^ assessed the feasibility of using serum LDH levels to predict postoperative pneumonia in a cohort study of 187 patients with aSAH. They found that LDH may be a useful biomarker in predicting postoperative pneumonia in patients with aSAH. More recently, in a retrospective cohort of 874 patients who underwent microsurgical clipping, Zheng et al.^17^ found an association between preoperative serum levels of LDH and mRS outcome at 3 months. The findings showed that a higher preoperative serum LDH level was associated with functional neurological outcomes at 3 months. In a study of 189 patients with spontaneous SAH in the emergency department, serum lactate is associated with short‐term in‐hospital mortality, but the study was hampered by the case–control design and limited follow‐up.^23^ However, the present study offers several advantages over previous studies. First, our study is large, with 3524 patients, which yielded great precision in estimates. Second, we used multivariable logistic regression and propensity score matching to minimize the bias from confounders. Our findings are still significant after including other important biomarkers which were not included in previous studies: troponin T,^16^, ^17^, ^23^ neutrophil count,^17^, ^23^ C‐reactive‐protein,^16^, ^17^ blood glucose,^17^ and procalcitonin.^16^, ^17^, ^23^ Third, the mortality data are accurate and complete, since death records were extracted from household registration data.^19^, ^20^ Fourth, this study also obtained continuous LDH levels for a month after admission, and thus, this study further assessed the impact of trajectory, median, and max LDH levels. Fifth, this study expanded the topic to evaluate the value of LDH in risk stratification beyond that of the original SAHIT model.

The mechanism of how high‐serum LDH level affects mortality in patients with aSAH is unknown. There are several possibilities that could explain the association between LDH and mortality. First, there is a tight link between LDH, necrosis, and ischemic pathologies. LDH is a cytoplasmic enzyme, and the release of LDH from the cytoplasm when the cell membrane is permeabilized after necrotic cell death.^24^ LDH is expressed in major organs, including the muscle, liver, and brain, and injuries in multiple organs secondary to stroke could lead to a significant change in serum LDH level.^25^ Secondly, necrosis and inflammation can induce each other and drive a local auto‐amplification loop that leads to exaggerated cell death and inflammation, leading to organ failure.^26^ Inflammation damages the brain–blood barrier and exacerbates injury of surrounding neurons and other cells in the brain.^27^, ^28^, ^29^ Our previous study supports that neutrophils, an inflammatory biomarker, are able to predict mortality after aSAH.^14^ Third, LDH might act as an index of the severity in aSAH. Our initial data showed high levels of LDH to be associated with high Hunt‐Hess scores.

LDH has been used as a late marker of myocardial damage for years. Cardiac complications, including the release of cardiac enzymes, are very common after subarachnoid hemorrhage and are associated with an increased risk of short‐term death. Thus, the association between LDH and short‐term mortality is potentially confounded by cardiac dysfunction. To explore this confounding relationship, we have adjusted Troponin T and history of coronary heart disease in the analyses. The association was still significant after adjusting for these confounders.

Our study is a retrospective study with inherent limitations. Retrospective analysis of electronic health record data is generally affected by unmeasured and residual confounders, missing data, and potential biases. We only analyzed all‐cause mortality and so did not examine the association of serum LDH level with cause‐specific mortality, which is not included in the Household Registration Administration System. Moreover, the trajectory of LDH needs to be interpreted with caution because of a lack of consistent process to measure LDH in this hospital. Future prospective researches are needed to confirm the findings. Finally, LDH occurs in all important human organs and lacks specificity to the central nervous system, and we collected serum LDH from blood, not the cerebrospinal fluid.

## Conclusion

In patients with aSAH, both baseline and longitudinal high‐serum LDH levels were associated with all‐cause mortality. The incorporation of LDH into a well‐established prediction model substantially improved both discrimination and reclassification of all‐cause mortality in patients with aSAH.

## Conflict of Interest

The authors declare that they have no conflict of interest.

## Author Contributions

Study concept: FF. Design: all authors. Acquisition, analysis, or interpretation of data: HD, PW, and XZ. Statistical analysis: HD and XZ. Drafting of the manuscript: HD and XZ. Critical revision of the manuscript for important intellectual content: all authors.

## Supporting information


**Table S1.** Multivariate analysis for mortality at 90 days.
**Table S2.** Logistic regression with least absolute shrinkage and selection operator analysis for mortality at 90 days.
**Table S3.** Associations between quartile of admission lactate dehydrogenase levels and mortality.
**Table S4.** In‐hospital complications stratified by quartile of lactate dehydrogenase levels.
**Table S5.** Reclassification for 90 days mortality.
**Table S6.** Associations between the clinical threshold of admission lactate dehydrogenase levels and mortality at 90 days.
**Figure S1.** Flow diagram of patients included in the cohort.
**Figure S2.** Relationship between lactate dehydrogenase and 90 days mortality in patients with aneurysmal subarachnoid hemorrhage.
**Figure S3.** Subgroup analysis of the association between lactate dehydrogenase levels and mortality at 90 days.
**Figure S4.** Receiver operating characteristic curves for Subarachnoid Hemorrhage International Trialists and addition of lactate dehydrogenase values for mortality at 90 days.
**Figure S5.** Calibration curves depicting the predicted vs observed 90 days mortality using the full Subarachnoid Hemorrhage International Trialists prediction models with and without lactate dehydrogenase.

## Data Availability

Data supporting the findings of this study are available from the corresponding author on reasonable request.
