# Supplementary material for: Lactate dehydrogenase predicting mortality in patients with aneurysmal subarachnoid hemorrhage
Source: Ann Clin Transl Neurol. 2022 Aug 19;9(10):1565–73. doi: 10.1002/acn3.51650 (PMC9539376; doi:10.1002/acn3.51650)
Supplement: Supplementary file 1 — Table S1. Multivariate analysis for mortality at 90 days. Table S2. Logistic regression with least absolute shrinkage and selection operator analysis for mortality at 90 days. Table S3. Associations between quartile of admission lactate dehydrogenase levels and mortality. Table S4. In‐hospital complications stratified by quartile of lactate dehydrogenase levels. Table S5. Reclassification for 90 days mortality. Table S6. Associations between the clinical threshold of admission lactate dehydrogenase levels and mortality at 90 days. Figure S1. Flow diagram of patients included in the cohort. Figure S2. Relationship between lactate dehydrogenase and 90 days mortality in patients with aneurysmal subarachnoid hemorrhage. Figure S3. Subgroup analysis of the association between lactate dehydrogenase levels and mortality at 90 days. Figure S4. Receiver operating characteristic curves for Subarachnoid Hemorrhage International Trialists and addition of lactate dehydrogenase values for mortality at 90 days. Figure S5. Calibration curves depicting the predicted vs observed 90 days mortality using the full Subarachnoid Hemorrhage International Trialists prediction models with and without lactate dehydrogenase. [file ACN3-9-1565-s001.docx]

# Appendix

**eTable 1 Multivariate Analysis for mortality at 90 days**

**eTable 2 Logistic regression with LASSO Analysis for mortality at 90 days**

**eTable 3 Associations Between Quartile of admission LDH Levels and mortality**

**eTable 4 In-hospital Complications Stratified by Quartile of LDH Levels**

**eTable 5 Reclassification for 90 days mortality**

**eTable 6 Associations Between clinical threshold of admission LDH Levels and mortality at 90 days**

**eFigure 1. Flow diagram of patients included in the cohort.**

**eFigure 2. Relationship between LDH and 90 days mortality in patients with aSAH.**

**eFigure 3. Subgroup analysis of association between LDH levels and mortality at 90 days.**

**eFigure 4 ROC curves for SAHIT and addition of LDH values for mortality at 90 days.**

**eFigure 5. Calibration curves depicting the predicted vs observed 90 days mortality using the full SAHIT prediction models with and without LDH.**

##### eTable 1 Multivariate Analysis for mortality at 90 days

| Characteristics | Unadjusted | | Multivariable Regression Adjustment | |
| --- | --- | --- | --- | --- |
|  | OR (95% CI) | P | OR (95% CI) | P |
| Demographics |  |  |  |  |
| Age, year, mean | 1.02(1.01-1.03) | <0.001 | 1.01(1.00-1.02) | 0.076 |
| Female | 1.34(1.08-1.65) | 0.007 | 1.34(1.04-1.71) | 0.21 |
| Smoking |  |  |  |  |
| Ever Smoking | 1.38(0.77-2.45) | 0.277 | NA | NA |
| Never Smoking | 1.17(0.68-2.02) | 0.569 | NA | NA |
| Alcohol abuse | 1.14(0.88-1.46) | 0.319 | NA | NA |
| Medical history, n (%) |  |  |  |  |
| Hypertension | 1.17(0.93-1.47) | 0.191 | NA | NA |
| Diabetes | 1.56(1.06-2.28) | 0.024 | 0.73(0.44-1.19) | 0.203 |
| Coronary heart disease | 1.11(0.58-2.10) | 0.753 | NA | NA |
| COPD | 1.56(1.10-2.22) | 0.013 | 1.29(0.85-1.94) | 0.230 |
| Chronic renal failure | 8.61(3.63-20.39) | <0.001 | 9.28(3.12-27.65) | <0.001 |
| Systolic blood pressure | 1.01(1.01-1.01) | <0.001 | 1.00(1.00-1.01) | 0.539 |
| Aneurysm characteristics |  |  |  |  |
| Location Posterior circulation | 1.33(1.04-1.71) | 0.026 | 1.05(0.78-1.41) | 0.747 |
| Size of aneurysm | 1.32(1.16-1.50) | <0.001 | 1.33(1.15-1.54) | <0.001 |
| Hemorrhagic characteristics |  |  |  |  |
| Fisher grade | 2.14(1.86- 2.46) | <0.001 | 1.51(1.29- 1.77) | <0.001 |
| Hunt & Hess grade | 2.83(2.52-3.18) | <0.001 | 1.65(1.43- 1.91) | <0.001 |
| External ventricular drain | 5.54(3.44-8.92) | <0.001 | 3.93(2.30-6.72) | <0.001 |
| operation |  |  |  |  |
| Clip | 0.18(0.14-0.22) | <0.001 | 0.20(0.16-0.26) | <0.001 |
| Coil | 0.19(0.13-0.27) | <0.001 | 0.23(0.15-0.35) | <0.001 |
| Baseline biomarker concentrations |  |  |  |  |
| Neutrophil count, ×10^9/L | 5.16(4.00-6.64) | <0.001 | 2.65(1.97-3.57) | <0.001 |
| Procalcitonin | 1.04(0.92-1.17) | 0.552 | NA | NA |
| C-reactive protein | 1.47(1.19-1.81) | <0.001 | 1.14(0.93-1.39) | 0.221 |
| Blood glucose, mmol/L | 8.79(6.33-12.20) | <0.001 | 4.27(2.79-6.55) | <0.001 |
| Troponin T, ng/L | 1.24(1.07-1.44) | 0.004 | 0.97(0.83-1.12) | 0.656 |
| LDH, U/L | 4.40(3.19-6.08) | <0.001 | 1.80(1.24-2.62) | 0.002 |

LDH: Lactate dehydrogenase

**eTable 2 Logistic regression with LASSO Analysis for mortality at 90 days**

| Characteristics | Logistic Regression with LASSO-penalty | |
| --- | --- | --- |
|  | OR (95% CI) | P |
| Demographics |  |  |
| Age, year, mean | NA | NA |
| Female | NA | NA |
| Smoking |  |  |
| Ever Smoking | NA | NA |
| Never Smoking | NA | NA |
| Alcohol abuse | NA | NA |
| Medical history, n (%) |  |  |
| Hypertension | NA | NA |
| Diabetes | NA | NA |
| Coronary heart disease | NA | NA |
| Chronic obstructive pulmonary disease | NA | NA |
| Chronic renal failure | 9.03(3.14-25.95) | <0.001 |
| Systolic blood pressure | NA | NA |
| Aneurysm characteristics |  |  |
| Location Posterior circulation | NA | NA |
| Size of aneurysm | 1.31(1.12- 1.52) | <0.001 |
| Hemorrhagic characteristics |  |  |
| Fisher grade | 1.55(1.32- 1.81) | <0.001 |
| Hunt & Hess grade | 1.67(1.45- 1.93) | <0.001 |
| External ventricular drain | 2.89(1.68- 4.98) | <0.001 |
| Operation |  |  |
| Clip | 0.22(0.17- 0.28) | <0.001 |
| Coil | 0.26(0.17- 0.39) | <0.001 |
| Baseline biomarker concentrations |  |  |
| Neutrophil count, ×10^9/L | 1.06(1.03- 1.09) | <0.001 |
| Procalcitonin | NA | NA |
| C-reactive protein | NA | NA |
| Blood glucose, mmol/L | 1.08(1.04- 1.13) | <0.001 |
| Troponin T, ng/L | 1.00(1.00- 1.00) | 0.125 |
| LDH, U/L | 1.48(1.02-2.14) | 0.041 |

##### eTable 3 Associations Between Quartile of admission LDH Levels and mortality

| Outcomes | LDH Levels ([U/L](https://www.youlai.cn/ask/B0CE92malSF.html)) | Events, n (%) | Unadjusted OR | P trend | Multivariable Regression adjusted OR | P trend | PSM adjusted OR | P trend |
| --- | --- | --- | --- | --- | --- | --- | --- | --- |
| Mortality at 90 days | <162 | 52/898(5.8%) | 1 [Reference] | <0.001 | 1 [Reference] | 0.022 | 1 [Reference] | <0.001 |
|  | 162-189 | 64/867(7.4%) | 1.30(0.89-1.89) |  | 0.91(0.60-1.40) |  | 0.86(0.55-1.34) |  |
|  | 190-228 | 105/881(11.9%) | 2.20(1.56-3.11) |  | 1.06(0.70-1.61) |  | 1.16(0.75-1.78) |  |
|  | >228 | 187/878(21.3%) | 4.40(3.19-6.08) |  | 1.60(1.08-2.37) |  | 1.78(1.16-2.73) |  |
| Mortality at 180 days | <162 | 64/1017(6.3%) | 1 [Reference] | <0.001 | 1 [Reference] | 0.035 | 1 [Reference] | <0.001 |
|  | 162-189 | 92/953(9.7%) | 1.41(1.00-2.00) |  | 1.03(0.70-1.51) |  | 0.94(0.63-1.40) |  |
|  | 190-228 | 124/1000(12.4%) | 2.04(1.47-2.83) |  | 1.01(0.68-1.51) |  | 1.14(0.76-1.71) |  |
|  | >228 | 213/968(22%) | 4.15(3.07-5.63) |  | 1.58(1.09-2.29) |  | 1.66(1.11-2.49) |  |
| Mortality at one year | <162 | 69/898(7.7%) | 1 [Reference] | <0.001 | 1 [Reference] | 0.006 | 1 [Reference] | <0.001 |
|  | 162-189 | 88/867(10.1%) | 1.36(0.98-1.89) |  | 1.00(0.69-1.44) |  | 0.95(0.65-1.38) |  |
|  | 190-228 | 133/881(15.1%) | 2.14(1.57-2.90) |  | 1.12(0.77-1.63) |  | 1.19(0.81-1.75) |  |
|  | >228 | 229/878(26.1%) | 4.24(3.18-5.65) |  | 1.67(1.17-2.37) |  | 1.85(1.27-2.70) |  |
| Mortality at two years | <162 | 79/898(8.8%) | 1 [Reference] | <0.001 | 1 [Reference] | <0.001 | 1 [Reference] | <0.001 |
|  | 162-189 | 101/867(11.6%) | 1.37(1.00-1.86) |  | 1.04(0.73-1.47) |  | 1.05(0.74-1.49) |  |
|  | 190-228 | 159/881(18%) | 2.28(1.71-3.04) |  | 1.26(0.89-1.78) |  | 1.39(0.97-2.00) |  |
|  | >228 | 262/878(29.8%) | 4.41(3.36-5.79) |  | 1.69(1.21-2.35) |  | 1.83(1.28-2.62) |  |
| Mortality at longest follow-up | <162 | 105/898(11.7%) | 1 [Reference] | <0.001 | 1 [Reference] | 0.003 | 1 [Reference] | <0.001 |
|  | 162-189 | 136/867(15.7%) | 1.41(1.07-1.85) |  | 1.08(0.80-1.47) |  | 1.09(0.80-1.49) |  |
|  | 190-228 | 209/881(23.7%) | 2.35(1.82-3.03) |  | 1.47(1.09-2.00) |  | 1.55(1.13-2.14) |  |
|  | >228 | 317/878(36.1%) | 4.27(3.34-5.46) |  | 1.84(1.36-2.49) |  | 1.93(1.40-2.67) |  |

##### LDH: Lactate dehydrogenase

##### eTable 4 In-hospital Complications Stratified by Quartile of LDH Levels

| Outcomes | LDH Levels ([U/L](https://www.youlai.cn/ask/B0CE92malSF.html)) | Events, n (%) | Unadjusted OR | P trend | Multivariable Regression adjusted OR | P trend | PSM adjusted OR | P trend |
| --- | --- | --- | --- | --- | --- | --- | --- | --- |
| Pneumonia | <162 | 152/898(16.9%) | 1 [Reference] | <0.001 | 1 [Reference] | 0.001 | 1 [Reference] | <0.001 |
|  | 162-189 | 191/867(22%) | 1.39(1.09-1.76) |  | 1.18(0.90-1.54) |  | 1.31(1.00-1.71) |  |
|  | 190-228 | 226/881(25.7%) | 1.69(1.34-2.13) |  | 1.16(0.88-1.52) |  | 1.24(0.93-1.65) |  |
|  | >228 | 332/878(37.8%) | 2.98(2.39-3.72) |  | 1.93(1.48-2.54) |  | 1.63(1.21-2.19) |  |
| Intracranial infection | <162 | 93/898(10.4%) | 1 [Reference] | 0.012 | 1 [Reference] | 0.18 | 1 [Reference] | 0.835 |
|  | 162-189 | 82/867(9.5%) | 0.90(0.66-1.24) |  | 0.80(0.57-1.13) |  | 0.81(0.57-1.15) |  |
|  | 190-228 | 100/881(11.4%) | 1.11(0.82-1.49) |  | 0.78(0.55-1.11) |  | 0.87(0.60-1.26) |  |
|  | >228 | 118/878(13.4%) | 1.34(1.01-1.79) |  | 0.76(0.53-1.08) |  | 0.90(0.61-1.34) |  |
| Urinary tract infection | <162 | 108/898(12%) | 1 [Reference] | <0.001 | 1 [Reference] | 0.005 | 1 [Reference] | <0.001 |
|  | 162-189 | 114/867(13.1%) | 1.11(0.84-1.47) |  | 1.08(0.80-1.46) |  | 1.09(0.79-1.50) |  |
|  | 190-228 | 128/881(14.5%) | 1.24(0.94-1.64) |  | 1.18(0.88-1.59) |  | 1.27(0.91-1.77) |  |
|  | >228 | 165/878(18.8%) | 1.69(1.30-2.20) |  | 1.44(1.07-1.95) |  | 1.38(0.98-1.95) |  |
| Bloodstream infection | <162 | 12/898(1.3%) | 1 [Reference] | 0.001 | 1 [Reference] | 0.002 | 1 [Reference] | <0.001 |
|  | 162-189 | 17/867(2%) | 1.48(0.70-3.11) |  | 1.35(0.61-2.98) |  | 1.20(0.52-2.80) |  |
|  | 190-228 | 22/881(2.5%) | 1.89(0.93-3.84) |  | 1.81(0.87-3.77) |  | 1.19(0.53-2.67) |  |
|  | >228 | 42/878(4.8%) | 3.71(1.94-7.09) |  | 3.15(1.56-6.37) |  | 3.44(1.37-8.63) |  |
| hydrocephalus | <162 | 70/898(7.8%) | 1 [Reference] | <0.001 | 1 [Reference] | 0.50 | 1 [Reference] | 0.413 |
|  | 162-189 | 70/867(8.1%) | 1.04(0.74-1.47) |  | 0.84(0.57-1.23) |  | 0.87(0.59-1.29) |  |
|  | 190-228 | 93/881(10.6%) | 1.40(1.01-1.93) |  | 0.93(0.64-1.35) |  | 0.92(0.63-1.36) |  |
|  | >228 | 129/878(14.7%) | 2.04(1.50-2.77) |  | 1.00(0.69-1.47) |  | 0.98(0.65-1.47) |  |
| Re-bleeding | <162 | 30/898(3.3%) | 1 [Reference] | 0.001 | 1 [Reference] | 0.267 | 1 [Reference] | 0.066 |
|  | 162-189 | 31/867(3.6%) | 1.07(0.64-1.79) |  | 0.95(0.56-1.62) |  | 1.00(0.56-1.78) |  |
|  | 190-228 | 46/881(5.2%) | 1.59(1.00-2.55) |  | 1.09(0.65-1.83) |  | 1.29(0.69-2.42) |  |
|  | >228 | 57/878(6.5%) | 2.01(1.28-3.16) |  | 1.35(0.80-2.28) |  | 1.25(0.69-2.26) |  |
| Delayed cerebral ischemia | <162 | 148/898(16.5%) | 1 [Reference] | 0.004 | 1 [Reference] | 0.007 | 1 [Reference] | 0.013 |
|  | 162-189 | 159/867(18.3%) | 1.14(0.89-1.46) |  | 1.09(0.85-1.40) |  | 1.11(0.84-1.46) |  |
|  | 190-228 | 167/881(19%) | 1.19(0.93-1.51) |  | 1.13(0.88-1.46) |  | 1.10(0.81-1.49) |  |
|  | >228 | 192/878(21.9%) | 1.42(1.12-1.80) |  | 1.33(1.02-1.73) |  | 1.29(0.93-1.77) |  |
| Seizures | <162 | 22/898(2.4%) | 1 [Reference] | <0.001 | 1 [Reference] | <0.001 | 1 [Reference] | 0.003 |
|  | 162-189 | 21/867(2.4%) | 0.99(0.54-1.81) |  | 0.94(0.51-1.72) |  | 0.83(0.41-1.66) |  |
|  | 190-228 | 33/881(3.7%) | 1.55(0.90-2.68) |  | 1.20(0.67-2.17) |  | 1.19(0.61-2.35) |  |
|  | >228 | 51/878(5.8%) | 2.46(1.48-4.08) |  | 2.36(1.32-4.23) |  | 1.91(0.98-3.70) |  |

##### LDH: Lactate dehydrogenase

##### eTable 5 Reclassification for 90 days mortality

| SAHIT model | SAHIT +LDH admission model | | | | | | | |
| --- | --- | --- | --- | --- | --- | --- | --- | --- |
|  | No death | | | | Death | | | |
|  | [0,0.15) | [0.15,0.3) | [0.3,1] | Reclassified, % | [0,0.15) | [0.15,0.3) | [0.3,1] | Reclassified, % |
| [0,0.15) | 2539 | 32 | 0 | 1 | 135 | 8 | 1 | 6 |
| [0.15,0.3) | 36 | 341 | 30 | 16 | 6 | 97 | 21 | 22 |
| [0.3,1] | 0 | 18 | 120 | 13 | 0 | 7 | 133 | 5 |

| SAHIT model | SAHIT +LDH median model | | | | | | | |
| --- | --- | --- | --- | --- | --- | --- | --- | --- |
|  | No death | | | | Death | | | |
|  | [0,0.15) | [0.15,0.3) | [0.3,1] | Reclassified, % | [0,0.15) | [0.15,0.3) | [0.3,1] | Reclassified, % |
| [0,0.15) | 2522 | 49 | 0 | 2 | 128 | 13 | 3 | 11 |
| [0.15,0.3) | 63 | 292 | 48 | 27 | 10 | 89 | 25 | 28 |
| [0.3,1] | 0 | 22 | 116 | 16 | 0 | 7 | 133 | 5 |

| SAHIT model | SAHIT +LDH max model | | | | | | | |
| --- | --- | --- | --- | --- | --- | --- | --- | --- |
|  | No death | | | | Death | | | |
|  | [0,0.15) | [0.15,0.3) | [0.3,1] | Reclassified, % | [0,0.15) | [0.15,0.3) | [0.3,1] | Reclassified, % |
| [0,0.15) | 2556 | 11 | 4 | 1 | 133 | 9 | 2 | 8 |
| [0.15,0.3) | 22 | 359 | 26 | 12 | 5 | 103 | 16 | 17 |
| [0.3,1] | 0 | 9 | 129 | 7 | 0 | 4 | 136 | 3 |

LDH: Lactate dehydrogenase; SAHIT: Subarachnoid Hemorrhage International Trialists (SAHIT)

##### eTable 6 Associations Between clinical threshold of admission LDH Levels and mortality at 90 days

|  | LDH (U/L) | Events, n (%) | Unadjusted OR | Multivariable Regression adjusted OR |
| --- | --- | --- | --- | --- |
| Continuous | Logarithmic transformations | NA | 6.45(4.08-10.19) | 1.98(1.30-3.02) |
| Clinical threshold | ≼333 | 357/3372(10.6%) | 1 [Reference] | 1 [Reference] |
|  | >333 | 51/153(33.6%) | 4.26(2.99-6.08) | 1.63(1.05-2.53) |
| Quartile | <162 | 52/898(5.8%) | 1 [Reference] | 1 [Reference] |
|  | 162-189 | 64/867(7.4%) | 1.30(0.89-1.89) | 0.91(0.60-1.40) |
|  | 190-228 | 105/881(11.9%) | 2.20(1.56-3.11) | 1.06(0.70-1.61) |
|  | >228 | 187/878(21.3%) | 4.40(3.19-6.08) | 1.60(1.08-2.37) |

LDH: Lactate dehydrogenase


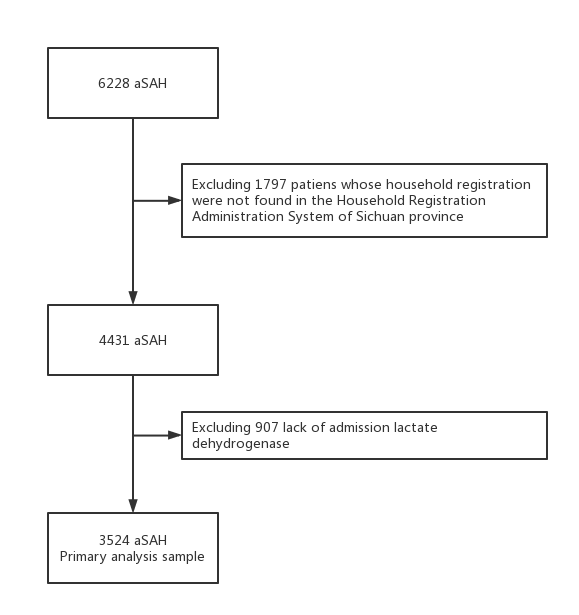


**eFigure 1 Flow diagram** **of patients included in the cohort**


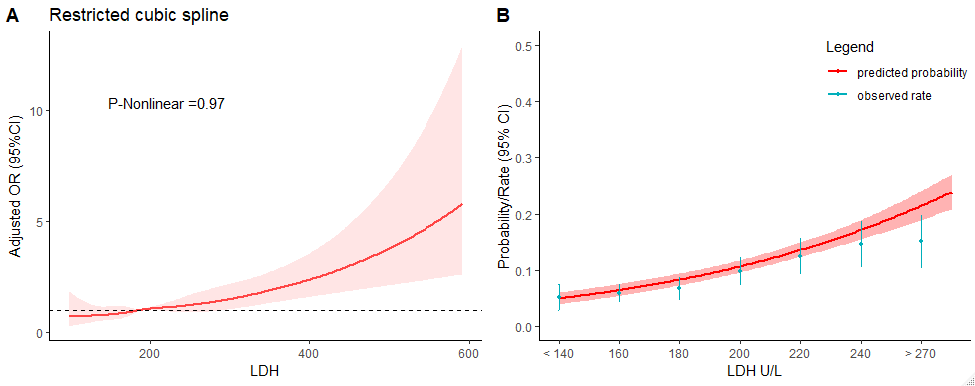


**eFigure 2. Relationship between lactate dehydrogenase and 90 days mortality in patients with aSAH.**

**
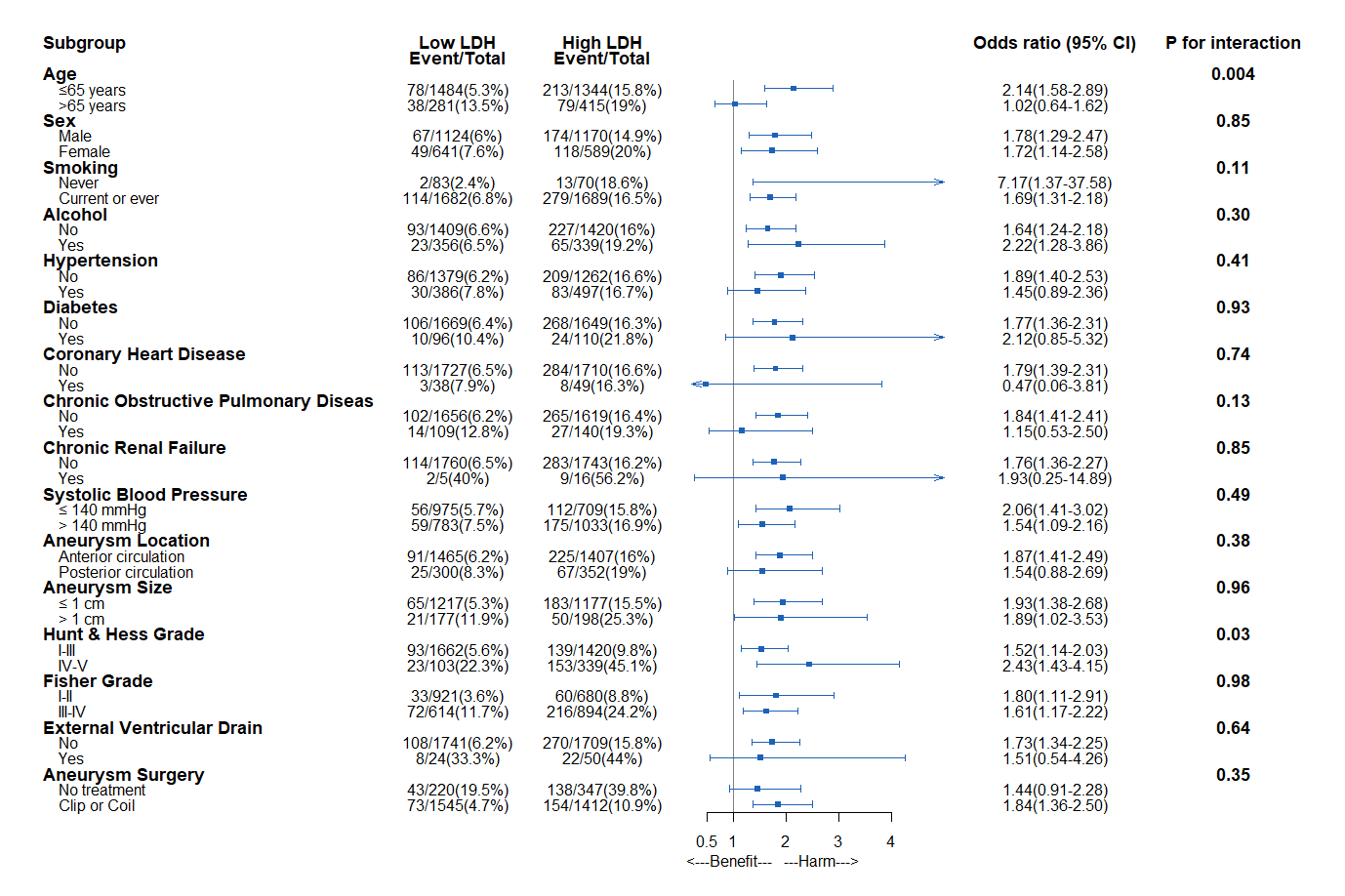
 eFigure 3. Subgroup analysis of association between LDH levels and mortality at 90 days**


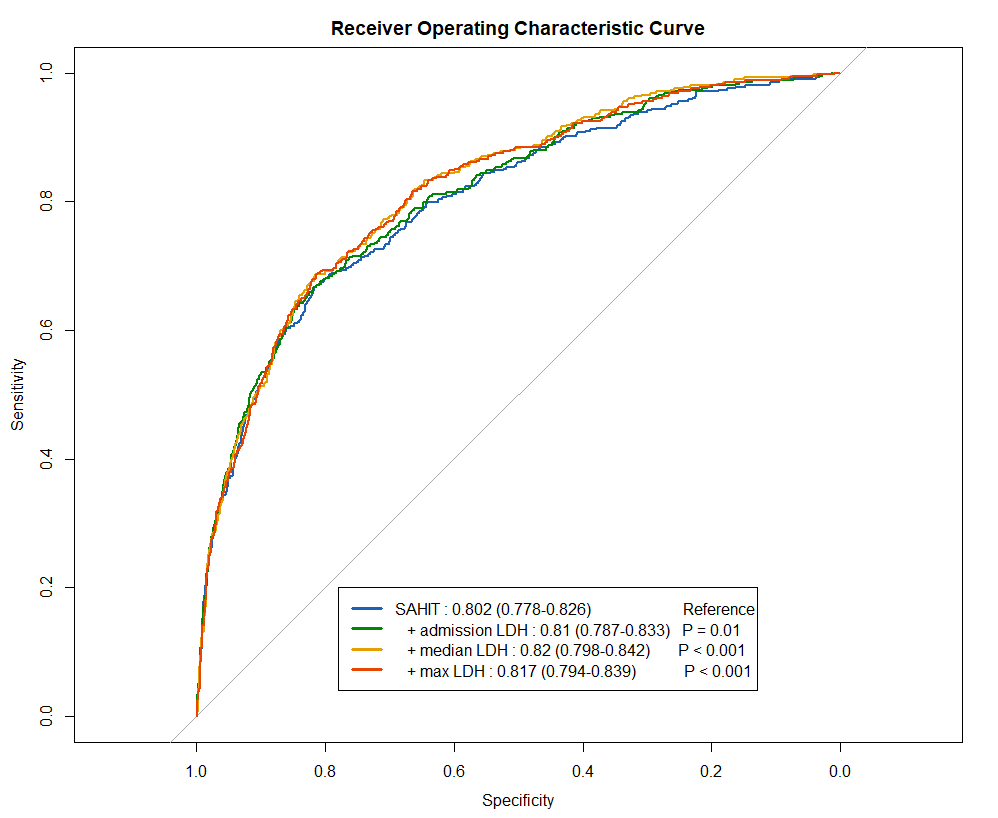
**eFigure 4 ROC curves for SAHIT and addition of LDH values for mortality at 90 days**

LDH: Lactate dehydrogenase; SAHIT: Subarachnoid Hemorrhage International Trialists (SAHIT)


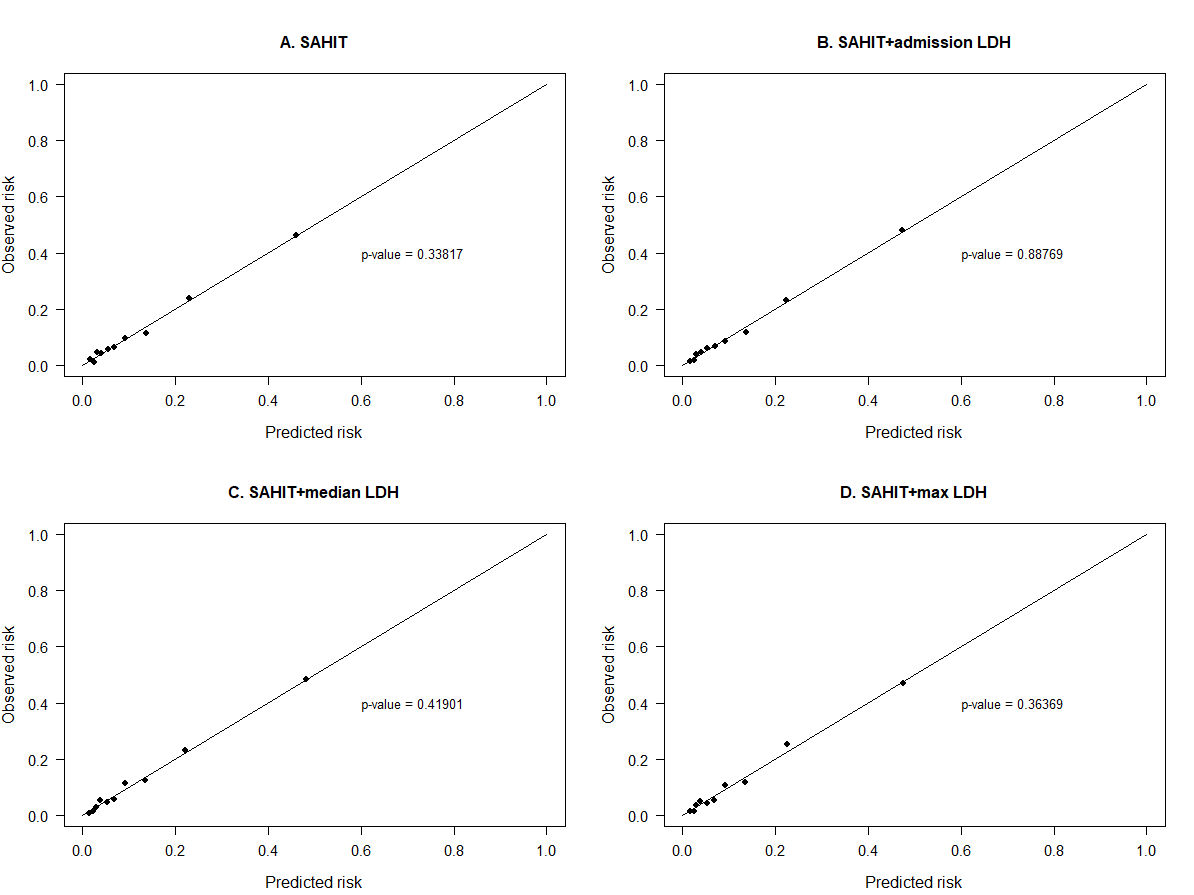


**eFigure 5. Calibration curves depicting the predicted vs observed 90 days mortality using the full SAHIT prediction models with and without LDH**

LDH: Lactate dehydrogenase; SAHIT: Subarachnoid Hemorrhage International Trialists (SAHIT)
